# Listen protect connect for traumatized schoolchildren: a pilot study of psychological first aid

**DOI:** 10.1186/2050-7283-1-26

**Published:** 2013-11-27

**Authors:** Marizen Ramirez, Karisa Harland, Maisha Frederick, Rhoda Shepherd, Marleen Wong, Joseph E Cavanaugh

**Affiliations:** Department of Occupational and Environmental Health, University of Iowa, 105 S. River St. #318, Iowa City, IA 52242 USA; Injury Prevention Research Center, University of Iowa, Iowa City, IA USA; Cedar Rapids Community School District, Cedar Rapids, IA USA; School of Social Work, University of Southern California, Los Angeles, CA USA; Department of Biostatistics, University of Iowa, Iowa City, IA USA

**Keywords:** Post-traumatic stress disorders, Intervention studies, Schools, Child

## Abstract

**Background:**

Listen Protect Connect (LPC), a school-based program of Psychological First Aid delivered by non-mental health professionals, is intended to support trauma-exposed children. Our objective was to implement LPC in a school setting and assess the effectiveness of LPC on improving psychosocial outcomes associated with trauma.

**Methods:**

A pilot quasi-experiment was conducted with middle school children self-identified or referred to the school nurse as potentially exposed to stressful life experiences. LPC was provided to students by the school nurse, and questionnaires were administered at baseline, 2-, 4- and 8-weeks to assess life stressors, symptoms of post-traumatic stress disorder and depression, social support, and school connectedness. A total of 71 measurements were collected from 20 children in all. Although a small sample size, multiple measurements allowed for multivariable mixed effects models to analyze changes in the repeated outcomes over time.

**Results:**

Students who received the intervention had reduced depressive and posttraumatic stress symptoms from baseline throughout follow-up period. Total social support also increased significantly from baseline through 8-weeks, and school connectedness increased up to 4-weeks post-intervention.

**Conclusions:**

This study demonstrates the potential of LPC as a school-based intervention of Psychological First Aid. Future randomized trials of LPC are needed, however.

## Background

Trauma is defined as incidents experienced, witnessed or learned about that 1) involve “actual or threatened death or serious injury, or other threat to one or another’s physical integrity” and 2) elicit intense “fear, helplessness or horror” (American Psychiatric Association 2000)”. Trauma is common in youth, impacting as many as 80% of children worldwide (Sharma-Patel et al. 2011). In a US-based longitudinal study, 68.8% of children were exposed to one or more traumatic events by their 16^th^ birthday (Copeland et al. 2007). Children experience a variety of traumas, including learning about traumatic experiences of relatives or friends (62%), sudden death of friends or relatives (60%), assaults (38%), motor vehicle crash (28%), and natural disasters (i.e., tornados, fire, flood or earthquake) (17%) (Breslau et al. 1998). Although extremely rare, school shootings, such as the recent shooting at Sandy Hook Elementary School in Newtown, Connecticut, also represent the types of trauma that may directly impact children.

Exposure to trauma may trigger adverse psychological responses, of which post-traumatic stress disorder (PTSD) and depression are most prominent (Kenardy et al. 2006). Studies show great variability in the rates of PTSD, depending on the type of, severity of, and time elapsed since a traumatic event. Between 23-70% of children exposed to natural disasters and 10-80% of children witnessing violence display symptoms of PTSD (McDermott et al. 2005; Neuner et al. 2006; Vernberg et al. 1996; Pynoos 1993; Goenjian et al. 2005; Nader et al. 1990; Hoven 2005; Ahmad et al. 2000). PTSD also tends to co-occur with other types of psychiatric disorders, particularly depression. Thirty-seven to 47% of PTSD diagnosis in children is accompanied by a diagnosis of depression. In an urban population of 1,007 youths exposed to violence, 23.6% developed PTSD and among those, 36.6% had major depression (Breslau et al. 1991). Among child witnesses of violent crime with PTSD, 47% were also found to be diagnosed with depression in comparison to 16% without PTSD (Muesar and Taub 2008).

Schools are a place where children often exhibit signs of trauma-related distress, and can therefore serve as a successful point of contact and treatment (President’s New Freedom Commission on Mental Health 2003). Currently, the state of school mental health practice focuses on referring students who are at high risk for developing mental health disorders to a school psychologist for individual care (Dowdy et al. 2010; Cash & Nealis 2004). Of therapeutic methods used in schools, Cognitive Behavior Therapy (CBT) and trauma/grief-informed psychotherapy have been found to effectively reduce symptoms of depression and PTSD among trauma-exposed youth (Goenjian et al. 2005; Stein et al. 2003; Layne 2001). CBT and psychotherapy are both time-intensive modalities supported by professional mental health clinicians and intended for use among individuals with full-blown PTSD (symptoms after 30 days).

An important gap of service exists in the areas of triage and early intervention, which are the critical first steps that can direct trauma-exposed students to advanced care. The most common early intervention treatment in school mental health practice is Psychological Debriefing, a community-based early psychological intervention delivered to trauma exposed individuals. It was initially concluded as effective in reducing an array of psychopathology symptoms (Flannery and Everly 2004). However, recent randomized controlled trials conducted among adults, children and adolescents demonstrated that Psychological Debriefing failed to improve outcomes when compared with a control group (Stallard et al. 2006; Hobbs et al. 1996). As a result of these contradicting findings, the Task Force on Community Preventive Services recommended against the use of this therapy among trauma-exposed children and adolescents (Wethington et al. 2008). To date, there are no evidence-based triage and early interventions delivered by non-mental health professionals for trauma-exposed students.

To address this service gap, Listen, Protect & Connect (LPC) was developed as an intervention program of Psychological First Aid. Psychological First Aid, which is analogous to physical First Aid, involves post-trauma contact and engagement, safety and comfort, stabilization, information gathering, practical assistance, connection with social supports, information on coping support and linking to services (Ruzak et al. 2007). Informed by research on posttraumatic resilience (Kataoka et al. 2012; Wong 2008), LPC was initially designed for delivery by a non-professional to provide information, education, comfort and support to traumatized youth after a community disaster or emergency. However, the elements of LPC could also be used to support children impacted by personal traumas. The effectiveness of LPC in improving children’s recovery from trauma has not been scientifically evaluated. Hence, we began a small-scale study of LPC delivered by school nurses in a school district in Iowa (US). Our implementation and outcome evaluation of LPC was conducted to (1) describe the acceptability and barriers of this program, and (2) measure the extent to which LPC reduces symptoms of psychological distress and improves social support and school connectedness.

## Methods

### Participants

A pilot quasi-experiment was conducted with 20 middle and high school students enrolled in four middle and two high schools from a single urban school district in the Midwest from May 2009 through 2011. These subjects were recruited from two consecutive school years.

Our year one eligible population was comprised of students directly impacted by the 2008 Great Flood of Iowa, identified from the school district’s list of relocated students. Due to IRB delays, time required for training and district approvals, eligible students were recruited approximately 10 months after the flood.

To increase our sample size in year two, additional students potentially traumatized by other types of traumas (such as violence or death of a loved one) were recruited from the same schools involved in year one. A number of indicators were used to identify these students, based on prior research on factors associated with trauma (Caffo and Belaise 2003). To be eligible, students either 1) had to be seen at the nurse’s office for nonspecific physical symptoms (e.g., vague headaches, stomachaches) or behavioral problems at least 1×/week for three consecutive weeks or 2×/week for two weeks, 2) had to have reported a personal trauma or expressed distress to the nurse or school staff, or 3) had to have 3–5 consecutive days of unexcused absences.

Eligible students and their parent(s) were mailed an introductory letter about the study and asked to return a self-addressed postcard if interested in participating. Interested families were mailed an information sheet, informed assent/consent documents and an enrollment and contact form. Our passive recruitment efforts yielded a sample of 8 flood-affected students and 12 students with a history of individual trauma. This study was approved by the University of Iowa Institutional Review Board. We obtained parental consent and child assent for participation.

### Procedure

Nurses from the six middle and high schools received three hours of training in LPC by the developer (M. Wong) and Principal Investigator (M. Ramirez) in year one. Participants were provided basic information on trauma and its psychological impacts on children. The three required steps of Listen, Protect, and Connect were each described in detail. Manuals, worksheets, and pocket cards summarizing these key steps were provided; nurses participated in role playing to increase familiarization with the steps. In year two, a two hour refresher course was provided to review LPC steps and materials with a focus on individual traumas such as interpersonal violence and injury.

After obtaining assent and consent, baseline questionnaires were distributed to the students by the University of Iowa research team online or in-person. To confirm exposure to trauma, students were asked to report the types of traumas experienced, witnessed, or learned about through the Life Events Checklist (LEC), a scale with adequate reliability and validity. Respondents reported their traumatic experiences on a 5-point scale (1 = happened to me, 2 = witnessed it, 3 = learned about it, 4 = not sure, and 5 = does not apply) (Gray et al. 2004). All students had personally experienced, witnessed or learned about a traumatic event therefore meeting PTSD Criterion A.

Within one week of baseline survey completion, an LPC session was scheduled with the school nurse. After completion of the LPC session, both the nurse and student completed LPC session evaluation forms. Follow-up questionnaires were completed by the student at 2-, 4- and 8-weeks following the initial LPC session.

### Intervention

*Listen Protect Connect* (LPC) is composed of three basic steps designed to specifically target PTSD symptomatic reactions (Kataoka et al. 2012; Wong 2008).

#### Listen step 1

Interventionists use reflective listening skills and non-invasive questions to elicit responses about a student’s specific traumatic experiences. For example, the interventionist asks the students “How, What, or Tell me more…” questions to begin an open dialogue of the student’s concerns.

#### Protect step 2

The interventionist conducts a brief screener of non-specific distress using the six-item K6 screener (Furukawa et al. 2003). The interventionist is taught to identify cognitive, physiological, and psychological reactions to trauma, and engages in open discussion with the student about their fears and worries. Through assessment and honest discussion, the interventionist “protects” students by identifying potentially high risk children who score high on the K6 screener or reveal maladaptive reactions to trauma. The interventionist is therefore equipped with critical information indicating need for additional services. During this step, as concerns and worries surface, the interventionist engages in open discussion about the crisis and actions taken by schools, families and schools to keep the traumatized child safe. This includes discussions about school safety protocols, support provided by parents and families or by the local community or school, or assistance provided by professionals such as counselors and nurses on campus.

#### Connect step 3

The PFA interventionist uses information from Steps 1 and 2 to identify students who may be at risk for potential distress. The interventionist then facilitates access to resources and advanced mental health care. Furthermore, the interventionist encourages the student to re-connect with friends, family and to re-engage in previously enjoyed activities. LPC is a flexible program that could be implemented repeatedly and as short or as long as the interventionist and student desire.

### Implementation evaluation with nurses and students

Using post-session evaluation forms, school nurses reported the number and length of each LPC session, as well as the ease or difficulty in completing each LPC step using a 5-point scale where 1 is very easy to 5 is very difficult. School nurses also reported use of the program materials (worksheets, manual, screener, pocket card) and how helpful these materials were during the delivery of the session (1 = not at all to 5 = very helpful). Using a similar 5-point scale (1 = not at all to 5 = very comfortable), students were asked to provide comfort level while communicating with the school nurse, and comfort with the length of the LPC session. Both the nurse and student were asked to report their perceived helpfulness of the LPC session (1 = not at all helpful to 5 = very helpful). School nurses described perceived barriers and suggestions to improve the program.

### Outcome evaluation

#### Instruments

We collected the following measures from students in baseline and follow-up questionnaires:

The modified Child PTSD Symptom Scale is the 17-item child version of the adult posttraumatic diagnostic scale with scores ranging from 0 to 51 (Chronbach α = 0.89) (Foa et al. 2001). A cut point of 14 was used to classify children as symptomatic for PTSD (Stein et al. 2003).

To measure depressive symptoms, we utilized the Center for Epidemiologic Studies Depression Scale (CES-D), a 20-item self-rating scale assessing frequency of symptoms. A child with a score of 16 or higher was categorized as displaying depressive symptoms (Radloff 1977).

The Multidimensional Scale of Perceived Support (MSPSS) is a 12-item scale measuring perceived social support from family, friends and a significant other (Chronbach α = 0.93) (Zimet et al. 1990; Canty-Mitchell and Zimet 2000; Bruwer et al. 2008).

To assess the extent to which students feel connected to their school, we used selected items from the Healthy Kids Resilience Measure of School Connectedness that measure students’ perceived connectedness with adults at their school and the strength of these relationships (Constantine et al. 1999). All items in this scale showed strong internal consistency (Cronbach α = 0.87).

Age, gender and ethnicity, potential confounders identified from prior research, were also collected from students at baseline (Davis and Siegel 2000).

### Data analysis

For the implementation evaluation, we performed descriptive analysis to describe the ease/difficulty and perceived helpfulness of LPC steps and materials. For open-ended questions about barriers, we used content analysis to identify and create categories of themes.

For our outcome evaluation, we analyzed changes in psychological symptoms, social support and school connectedness over time. To control for the correlation among longitudinal responses collected on the same student and among responses collected within the same school, we first fit hierarchical mixed effects linear regression models that included random effects to induce clustering at both the student level and the school level. Compared with standard repeated measures ANOVA, the hierarchical mixed effects model is a more flexible approach to account for irregular time measurement points, missing observations and time-dependency (Gueorguieva and Krystal 2004). We fit our initial models with an autoregressive correlation structure at the student level to allow for the magnitude of the correlation between two measurements to depend on the time period between the measurements. (For example, observations taken at 2- and 4- weeks follow-up are assumed to be more highly correlated than measurements taken at 2- and 8-weeks follow-up.) At the school level, we employed an exchangeable correlation structure. However, based on the variance component estimates for these models, there was no evidence of school-level clustering. Therefore, we used a simpler mixed effects model that accounted only for the correlation among longitudinal responses collected on the same student. Age, gender, ethnicity and types of trauma were included in the model as covariates and potential confounders. Statistical significance was set at a p-value <0.05 but, given the small sample size, p-values of <0.10 are also documented as suggestive of change.

All study activities were approved by the University of Iowa Institutional Review Board. All data analysis was conducted using SAS® software, Version 9.2 of the SAS System for Microsoft, SAS Institute Inc., Cary, NC, USA.

## Results

### Description of subjects at baseline

A total of 20 students completed LPC sessions over two phases. Approximately, 80% of the students were male between the ages 12–17 years and represented grades 6^th^ through 11^th^ (Table 1). Twenty students completed baseline and 2-week follow-up questionnaires, 18 completed the 4-week follow-up, and 15 completed the final 8-week follow-up. There were a total of 71 repeated measures among the students.

**Table 1:** **Demographics and baseline symptomology of students experiencing trauma and receiving Psychological First Aid, Iowa, 2009–2010 (N = 20)**

	All (N = 20)	
Demographics	n (Col %)	Mean (SD)
**Age**		
12	7 (35.0)	
13	4 (20.0)	
14	3 (15.0)	
15	4 (20.0)	
≥16	2 (10.0)	
**Gender**		
Male	16 (80.0)	
Female	4 (20.0)	
**Race**		
White	7 (35.0)	
Hispanic	1 (5.0)	
African-American	1 (5.0)	
Asian/Pacific Islander	8 (40.0)	
Other race	3 (15.0)	
**Grade**		
6^th^	7 (35.0)	
7^th^	4 (20.0)	
8^th^	2 (10.0)	
9^th^	2 (10.0)	
10^th^	4 (20.0)	
11^th^	1 (5.0)	
**Symptoms**		
Depressive symptoms^1^	60% (n = 12)	23.5 (13.4)
PTSD symptoms^2^	55% (n = 11)	16.4 (12.8)
# of Traumas experienced^3^		
Happened to student		4.0 (2.0)
Student witnessed		2.4 (2.8)
Student learned about		2.1 (3.4)
**Social support** ^**4**^		
Overall		3.6 (1.0)
Family		3.5 (1.2)
Friends		3.7 (1.0)
Significant other		3.8 (0.9)
**School connectedness** ^**5**^		58.4 (10.7)

^1^Center for Epidemiologic Studies Depression Scale (CES-D, 20 item), range (0–60), score ≥16 considered exhibiting mild/moderate/major depressive symptoms.

^2^Post-Traumatic Stress Disorder Checklist (civilian), score ≥14 demonstrates showing PTSD symptoms.

^3^Life Events Checklist.

^4^Multidimensional Scale of Perceived Social Support, Overall = average of all 12 items, Subscales (family, friends, significant other) = average of 4 items within each subscale.

^5^Healthy Kids Resilience Measure.

At baseline, 60% (n = 12) and 55% (n = 11) of students were symptomatic for depression and PTSD, respectively. The average depressive (M, SD = 23.5, 13.4) and PTSD (M, SD =16.4, 12.8) symptoms scores exceeded the cut point for demonstrating clinical symptomology of these conditions (Table 1).

Prior to the intervention, students were neutral or somewhat agreed (mean = 3.6, range 1–5) to having individuals in their lives to support or help them. A moderate level of school connectedness was reported by the students (mean = 58.4, median = 52.5).

### Implementation evaluation

#### School nurse feedback to implementing LPC

The school nurse only provided one session of LPC to each student in the school nurse office. Sessions lasted on average 25 minutes long (range 10 to 40 minutes). Programmatic steps of LPC were somewhat to very easy to implement by school nurses during almost all sessions (Table 2). No nurses reported any steps being very difficult. Nurses indicated that the Protect tools were effective in assisting the students to “convey their issues” and determine solutions. The Connect step was reportedly a “great closing conversation” to discuss coping strategies and resources. One nurse reported that this step “helped the student to organize his feelings to know what he truly needed”. In only one session did the nurse report struggling through the Protect and Connect steps.

**Table 2:** **School Nurses’ feedback on the effectiveness and adaptability of Psychological First Aid steps during the sessions**

Did you use:	How easy or difficult were these steps? n (%)			
	Very easy	Somewhat easy	Neither easy nor difficult	Somewhat difficult
The Listen Step?	13 (65%)	5 (25%)	2 (10%)	0
The Protect Step?	11 (55%)	6 (30%)	2 (10%)	1 (5%)
The Connect Step?	9 (45%)	6 (30%)	4 (20%)	1 (5%)

(N = 20).

During most of the LPC sessions, nurses reported using intervention materials (e.g., worksheets, manual and pocket card) and found them to be somewhat to very helpful.

#### Student feedback

Ninety-five percent of the students thought the sessions lasted about the right amount of time. Sixty percent felt very comfortable speaking with the school nurse; only one student reported feeling very uncomfortable. The sessions were rated as somewhat helpful in dealing with the recent events in their lives by 40% of the students. Students reported enjoying the opportunity to “share my feelings with someone else” and having someone to “listen to my problems”. One student reported that the nurse was “awesome and helped me by talking and understanding me”.

#### Barriers

Although a largely successful intervention, two nurses reported the length of the sessions and the scheduling of sessions as barriers. One nurse reported difficulty in scheduling a time most convenient for the student. Furthermore, students experienced a range of traumatic experiences (e.g. bullying, difficulty socializing with peers, a shooting, flood), and this heterogeneity was somewhat challenging for nurses. Nurses were first trained in LPC in response to the flood, and two found it somewhat difficult to relate some intervention materials to the students’ current situation.

### Outcome evaluation

**Figure 1:**
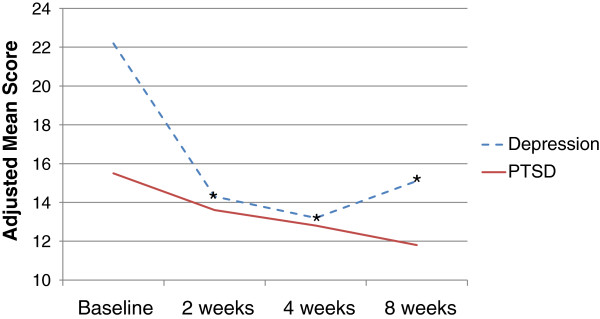
**Mean depressive and PTSD symptoms, Iowa PFA pilot, N = 71 measurements** ^**1**^ **.**

**Figure 2:**
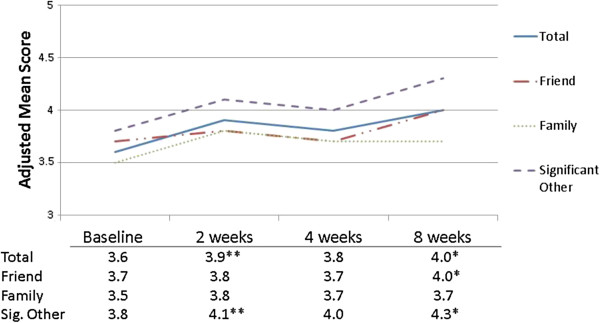
**Means for total social support and by source of social support, Iowa PFA pilot, N = 71 measurements** ^**1**^ **.**

**Figure 3:**
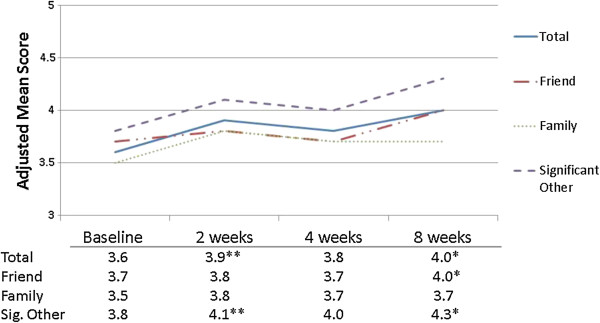
**Mean school connectedness, Iowa PFA pilot, N = 71 measuerment** ^**1**^ **.**

After controlling for students’ race, gender, sex and type of trauma (flood or individual trauma), a significant decline in depressive symptoms was seen from baseline to each follow-up period (Figure 1). The adjusted baseline mean depressive score was 22.2; this dropped to 14.3 (2-weeks, p < 0.01), 13.2 (4-weeks, p < 0.01) and increased just slightly to 15.2 (8-weeks, p < 0.01), all levels below the clinical cut point for depression. PTSD symptoms decreased 3.7 points from baseline to the 8-week follow-up, although this change was not statistically significant (range 15.5-11.8; p = 0.09). Total social support (Figure 2) increased from baseline to the 2-week follow-up (p = 0.08), and increased significantly from baseline to the 8-week follow-up (p < 0.01). The increase in average social support from significant others bordered on significance at 2-weeks (p = 0.09), but a strong, significant increase was seen by 8-weeks (p < 0.01). Students felt more connected to their school at 2- (mean = 63.8, p = 0.06) and 4-weeks (mean = 68.9, p < 0.01) than at baseline (mean = 58.6), but this relationship diminished by 8-weeks (Figure 3).

## Discussion

After recent tragedies, like the Boston Marathon Bombings, the Sandy Hook School Shooting and the many individual traumas experienced by students, school must quickly implement crisis intervention programs to support traumatized students. Psychological First Aid, one type of crisis intervention recommended by the National Child Traumatic Stress Network, is a promising approach but still lacks an evidence base.

Our pilot study is the first evaluation of a school-based specific form of Psychological First Aid, Listen Protect Connect (LPC), and our research showed that LPC was well-received by school interventionists and youth. Moreover, LPC has some promising evidence of effectiveness even in this pilot study, as treated youth had improved symptoms of psychological distress and increased school connectedness and social support.

Although marginally significant, the decrease in PTSD symptoms over time suggests an appropriate level of impact for an early intervention program delivered in a one-encounter session. As a first level of defense, LPC provides initial relief with a trusted adult at school. These findings are noteworthy, given that more complex school-based interventions report similar reductions in PTSD among children traumatized by violence, war or an earthquake (Goenjian et al. 2005; Stein et al. 2003; Layne 2001).

The significant decrease in depressive symptoms after treatment through LPC was particularly notable. On one hand, findings may have resulted from the natural regression to the mean, which cannot be ruled out given our lack of a control group. On the other hand, it is possible that LPC did indeed improve depressive symptoms. If LPC had no effect, depressive symptoms would have persisted at similar levels as baseline or increase, as demonstrated in prior studies of traumatized children who received no treatment (Goenjian et al. 2005; Stein et al. 2003). At the same time, a slight but notable increase in depressive symptoms was observed from 4 to 8 weeks. This also coincided with a decrease in school connectedness by 8 weeks, suggesting need for re-delivery of LPC with traumatized youth between 4–8 weeks after initial dosage. Despite these encouraging findings, clearly, a large trial is needed to gather more evidence of LPC’s effectiveness and its adequate dosage for maximal impact.

The LPC program teaches children to “re-establish social connectedness with family, teachers and peers”, and through improved social support, adopt positive coping and increase resilience (Kataoka et al. 2012; Wong 2008). Through these proposed mechanisms, it is feasible that the risk for developing PTSD and depression may be reduced. The increased social support and school connectedness reported among treated students were, therefore, consistent with our hypothesized effect of LPC.

Feedback from the school nurses who delivered LPC was overwhelmingly positive. Overall, LPC steps and related materials were perceived as helpful and easy to use during delivery. The few nurses who reported challenges in the delivery of LPC suggested minimal improvements in our training. Our revised manual will include specific response strategies to a variety of traumatic events and ideas for scheduling sessions so they are least disruptive (e.g., lunch time, after school). Students who received LPC also reported high acceptance of the intervention, with 40% of them likely to adopt coping strategies provided during the LPC session. These findings underscore a great potential for successful dissemination and implementation of LPC by schools.

LPC, a promising school-based post-traumatic intervention, is an alternative to practices currently in place and without an evidence base. For decades, school personnel, including nurses, have utilized Psychological Debriefing, a modality which has mixed evidence of effectiveness. If delivered as prescribed, Psychological Debriefing involves extensive probing within 48–72 hours of trauma exposure. In two randomized trials, debriefing did not improve post-trauma stress (Stallard et al. 2006; Hobbs et al. 1996), and it has been hypothesized that the deep probing involved in debriefing may lead to re-traumatization (Wethington et al. 2008). In contrast, other studies found that Psychological Debriefing significantly reduced PTSD symptoms among firefighters and alcohol use among soldiers (Mitchell et al. 1999; Deahl et al. 2000). As an alternative to debriefing, the LPC program uses reflective listening skills without deep probing. Our results show that LPC is not harmful to children; rather, the intervention facilitates identification of trauma and related distress, provision of initial relief, and referral to advanced care when necessary.

### Limitations

LPC is a program that is meant to be delivered soon after a traumatic experience. For our research study, it was not feasible to implement LPC after the flood due to IRB constraints and the time needed to adequately train school nurses in the LPC protocol. In the case of non-flood affected students, we were able to deliver LPC after a traumatized child was identified by the school nurse and informed consent was obtained. The time lag between exposure to trauma and treatment, which was not captured and controlled, may have impacted the effectiveness of LPC intervention and partially accounted for our inability to find a significant change in PTSD over time. Of note, even with this limitation, our analysis still suggested a decrease in symptomology. In fact, our analysis included students with both normative and elevated levels of depression and PTSD at baseline. We examined a sub-sample of students with elevated levels of depression and PTSD at baseline, and results were consistent. Therefore, based on these findings, LPC could potentially serve as a coping tool for all traumatized children, including those who might “fall through the cracks” with moderate distress.

For this pilot quasi-experiment, we also lacked a control group. To address this limitation, we adjusted for potential confounders such as age and gender. Ultimately, large-scale randomized trials are needed to add to LPC’s evidence base. Our small study sample had limited generalizability because recruitment was restricted to either self-referred children or students identified by the school nurse. Despite the small sample, we were able to conduct statistical tests through our repeated measures modeling framework. Our study is also prone to reporting bias due to the nature of self-administered questionnaires.

Finally, we examined LPC’s effect on different types of trauma – a situation also dictated by our small sample. Although we controlled for the type of traumatic experience (community disaster versus individual trauma), there may be unaccounted differing effects of the intervention on various individual traumas experienced by subjects (e.g., violent trauma, death of a loved one, severe injury). However, in practice, schools often encounter children with different types of traumatic experiences. An efficient approach to care is to provide school personnel with a repertoire of skills that can be modified to respond to different traumas. Lastly, we were unable to determine how many students sought formal mental health services following LPC – an important outcome of LPC. Our pilot study is the first evaluation of LPC, but future studies clearly are needed to address possible heterogeneity of effect, measure time lags from trauma to treatment, and post-LPC treatments.

## Conclusions

Trauma is commonly experienced by youth, and schools are a setting where trauma symptoms may surface and persist if untreated. Based on this pilot study, LPC, a form of Psychological First Aid delivered by school personnel, was found to be a promising response strategy. With reduced resources available for school-based mental health services, LPC is an efficient first-level of defense that identifies children in distress, provides initial support from a trusted adult, and links those most in need of advanced care.

## Abbreviations

LPC: Listen Protect Connect
PTSD: Post-traumatic stress disorder.
