# Erratum to: Listen protect connect for traumatized schoolchildren: a pilot study of psychological first aid

**DOI:** 10.1186/s40359-014-0045-0

**Published:** 2014-10-25

**Authors:** Marizen Ramirez, Karisa Harland, Maisha Frederick, Rhoda Shepherd, Marleen Wong, Joseph E Cavanaugh

**Affiliations:** Department of Occupational and Environmental Health, University of Iowa, 105 S. River St. #318, Iowa City, IA 52242 USA; Injury Prevention Research Center, University of Iowa, Iowa City, IA USA; Cedar Rapids Community School District, Cedar Rapids, IA USA; School of Social Work, University of Southern California, Los Angeles, CA USA; Department of Biostatistics, University of Iowa, Iowa City, IA USA

## Erratum

Due to an error in the publication process, this article (Ramirez et al. 2013) was published with an incorrect figure for Figure three (Figure 1 here).

**Figure 1:**
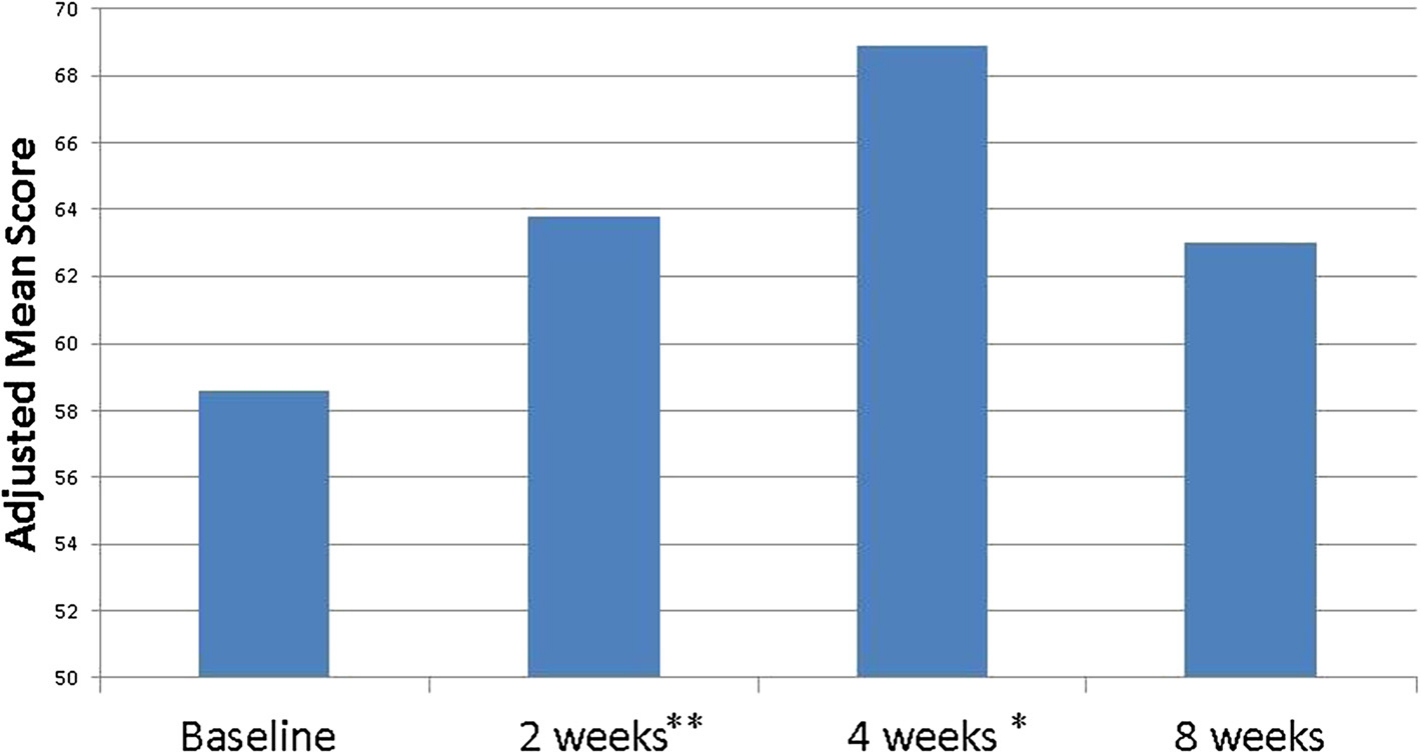
**Mean school connectedness, Iowa PFA Pilot, N = 71 measurements** ^**1**^ **.** ^1^Mixed effects model controls for student race, grade and sex, flood versus other trauma and correlation within a student. 71 measurements completed on 20 students. *p <0.05, **p<0.10 compared to baseline.

BioMed Central have now corrected this error and Figure three (Figure 1 here) can be found in this article. We apologise to the authors and the readers for any inconvenience caused.
